# Human rabies in China: evidence-based suggestions for improved case detection and data gathering

**DOI:** 10.1186/s40249-020-00672-9

**Published:** 2020-06-01

**Authors:** Hao Li, Jia-Jia Liu, Shu-Jun Ding, Liang Cai, Yun Feng, Peng-Cheng Yu, Shu-Qing Liu, Xue-Xin Lu, Xiao-Yan Tao, Wu-Yang Zhu

**Affiliations:** 1 Chinese Center for Disease Control and Prevention, Beijing, China; 2 National Health Commission of the People’s Republic of China, Key Laboratory of Biosafety, National Institute for Viral Disease Control and Prevention, Chinese Center for Disease Control and Prevention, 155 Changbai Road, Changping District, Beijing, 102206 China; 3 Shandong Center for Disease Control and Prevention, Jinan, Shandong China; 4 Hunan Center for Disease Control and Prevention, Changsha, Hunan China; 5 Yunnan Institute of Endemic Diseases Control and Prevention, Yunnan Provincial Key Laboratory for Zoonosis Control and Prevention, Dali, China

**Keywords:** Rabies, Human case, Diagnosis, Specimen, Detection

## Abstract

**Background:**

China still suffers heavily from rabies, although reported human cases continue to decrease year over year. There are far fewer laboratory-confirmed human cases than clinically diagnosed cases, which is a big problem that needs to be addressed. In this report, we summarize analyses of all specimens from human cases tested in our laboratory over the past 15 years, in order to promote laboratory diagnosis of rabies.

**Methods:**

From 2005 to 2019, a total of 271 samples from 164 suspected rabies cases were collected from local hospitals by the local Centers for Disease Control and Prevention (CDCs) in China. Saliva, cerebrospinal fluid (CSF), serum (blood) and urine were collected for ante-mortem diagnosis, and brain tissue, neck skin tissue and cornea were collected for post-mortem diagnosis. All of the specimens were tested by reverse transcription-polymerase chain reaction (RT-PCR), and brain tissues were also tested using fluorescent antibody test (FAT). The number of positive test results obtained using different fluids or tissues, and at different stages of the disease, were compared using a chi-square test and a more effective sampling program is recommended.

**Results:**

As the national reference laboratory for rabies surveillance in China, our laboratory has tested 271 samples from 164 suspected rabies cases collected by local CDCs since 2005. We found that saliva gave the highest number of positive test results (32%), compared with CSF and other fluids. We also found that serum or blood specimens collected in the last 3 days of life can test positive by RT-PCR.

**Conclusions:**

Serum or blood samples collected in the last 3 days of a patient’s life can be used to measure viral RNA, which means that serum samples, as well as saliva and CSF, can be used to detect viral RNA for anti-mortem diagnosis of rabies. Because of our findings, we have modified our “National Surveillance Project for Human Rabies”, by adding the collection and testing of serum samples from the end of the survival period. This will improve our national surveillance and laboratory diagnosis of human rabies.

## Background

Rabies is a fatal infectious viral disease that claims an estimated 59 000 human lives annually, mostly among underserved rural populations in Africa and Asia [1–3]. China is one of the countries affected by rabies and the number of human cases have fallen from 3300 in 2007 to 516 in 2017 under the government’s efforts [4–7].

In 2018, the World Health Organization (WHO) and other international agencies launched a global strategic plan to end human deaths from dog mediated rabies by 2030 [8]. China is actively responding to this call and is trying to improve capacity for the surveillance and control of rabies. Rabies, which is caused by viruses of the genus *Lyssavirus*, presents as an acute, progressive encephalitis [8, 9]. Clinical diagnosis of encephalitis can be difficult, and laboratory methods should be used to confirm a diagnosis whenever possible [8, 10]. In China, the low number of laboratory-confirmed cases of human rabies is an urgent problem that is hampering the elimination of rabies.

The WHO Expert Consultation on Rabies recommends that secretions, biological fluids (such as saliva, cerebrospinal fluid [CSF] and serum) and some tissues (such as skin biopsy samples, including hair follicles at the nape of the neck) should be used to diagnose rabies during life. Brain tissue is the preferred specimen for post-mortem diagnosis [8]. In China, however, it is very difficult to collect a brain specimen, or even a skin biopsy sample. CSF and serum samples are recommended for the detection of antibodies to the virus. Tests for neutralizing antibodies, such as the rapid fluorescent focus inhibition test (RFFIT), are, however, complicated and thus difficult to carry out in the local Centers for Disease Control and Prevention (CDCs) in China. Detection of viral RNA in bodily fluids is, therefore, the most important method for diagnosis of human rabies in China. The analysis of multiple different samples (e.g., skin, saliva, urine) at different stages of the disease has been shown to improve detection rate and is recommended for in life diagnosis [11].

As the national reference laboratory for rabies surveillance in China, our laboratory is responsible for training staff from the laboratories of provincial CDCs, and we have analyzed specimens of suspected rabies cases, collected by local CDCs, since 2005. In this report, we summarize the analyses of all specimens from human cases carried out by our laboratory over the past 15 years, and compare the rate of positive results from samples of different fluids or tissues and from different stages of the disease. Based on our results, we recommend a more effective sampling program, which will be a useful guide for improving the number of confirmed human rabies cases in China and other countries in a similar position.

## Methods

### Ethics statement

All human clinical specimens included in this study were collected by local CDCs for diagnostic confirmation of rabies in clinically suspected cases. The specimens were collected under the guidance of physicians and with the permission of the patients’ relatives. The specimens were then submitted to our laboratory for detection. All human samples were anonymized in this study. The program for collection of human specimens was approved by the Ethical Committee of the National Institute of Viral Disease Control and Prevention, China CDC.

### Specimens

Between 2005 and 2019, a total of 271 samples from 164 suspected rabies cases were collected from hospitals by local CDCs. The specimens included saliva, CSF, serum (blood) and urine for ante-mortem diagnosis, together with brain tissue, neck skin tissue and corneas for post-mortem diagnosis.

### Detections

Brain tissue, neck skin and cornea specimens were tested for rabies using FAT as previously described [12, 13]. Except for one cornea specimen, all of the samples, including fluids (saliva, CSF, serum and urine) and brain and skin tissues, were tested for the presence of viral RNA by nested RT-PCR as described earlier [14, 15]. Testing of serum specimens for viral RNA is not, however, recommended [8] and the WHO suggest collecting serum samples only for the detection of virus neutralizing antibodies in unvaccinated patients, but these tests are not very sensitive. The tests for neutralizing antibodies, such as RFFIT, are, however, very specialized and are difficult to popularize in local laboratories.

### Statistical analysis

Statistical analysis was carried out using SPSS version 25 (IBM Corp. Armonk, NY, USA), and a chi-square test was used to compare the positive test rate among the different types of samples (Table 1). The data will help us to decide whether sampling a particular fluid or tissue gives a higher positive detection rate and will thus be a better choice for diagnosis.

**Table 1:** The detection results of different type samples

	Samples	Total numbers	Positive numbers	Positive rate (%)
Anti-mortem	Saliva	125	40	32.0
	Urine	14	0	0.0
	Cerebrospinal fluid	19	4	21.1
	Serum (Blood)	85	9	10.6
Post-mortem	Brain tissue	23	23	100.0
	Neck skin tissue	4	2	50.0
	Cornea tissue	1	0	0.0

## Results

### Distribution of samples

**Fig. 1:**
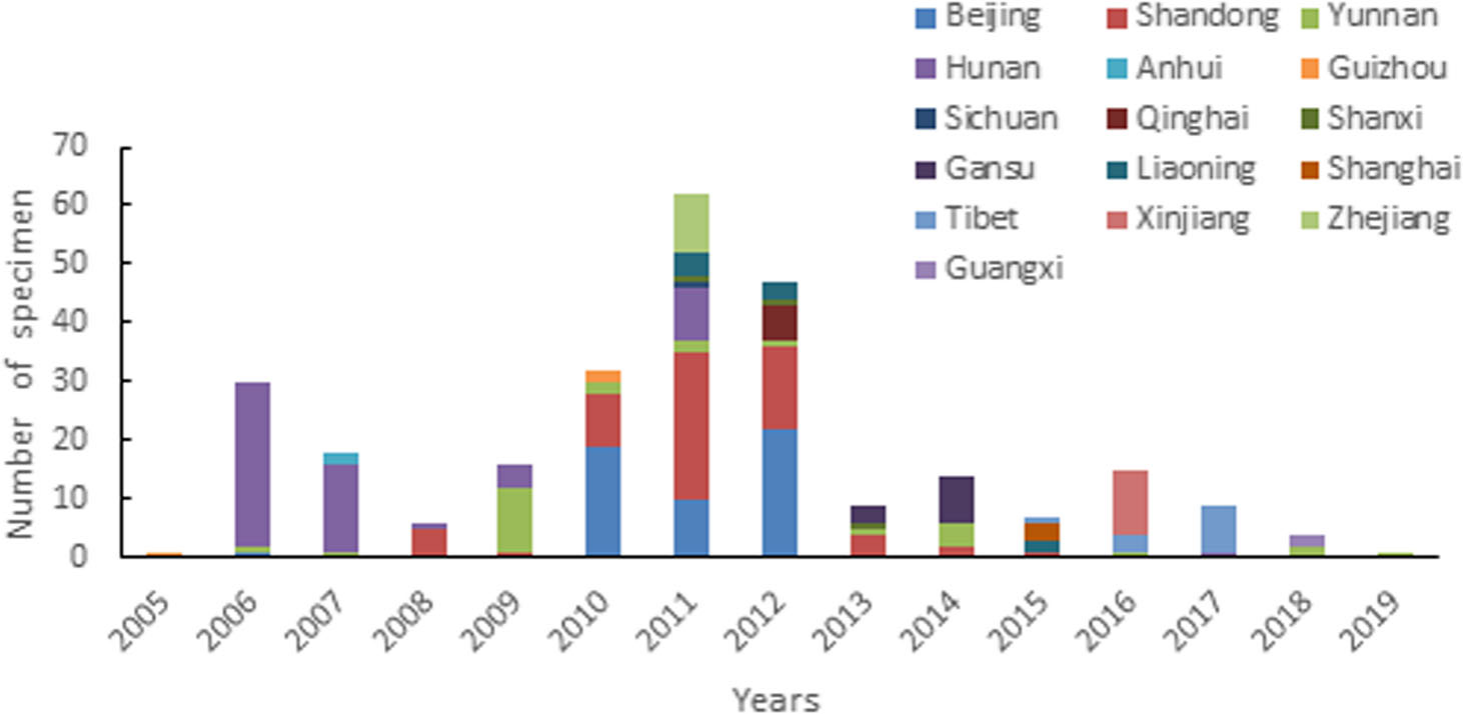
Numbers of specimens collected annually from different provinces

In total, 271 samples were collected over the past 15 years (Fig. 1). Only one sample was collected in 2005, with a slight increase in samples in 2006, followed by reduced sample numbers from 2007 to 2009. The largest numbers of samples were collected between 2010 and 2012, with relatively low and stable numbers over the last eight years. A total of 16 provinces submitted specimens, either continually or intermittently (Fig. 1). The largest numbers of samples were collected in 2011 (from eight provinces) and in 2012 (from six provinces). In other years, samples were obtained from 1 to 4 provinces.

Most samples were collected in Shandong, Hunan, Beijing and Yunnan, with about ten specimens from Tibet, Xinjiang, Gansu, Zhejiang and Liaoning. Six samples were collected in Qinghai, and only 1–3 samples were submitted from Guizhou, Shanxi, Shanghai, Guangxi, Anhui and Sichuan.

### Analysis of specimens

All samples were analyzed using the nested RT-PCR method for detection of viral RNA. Brain and skin tissues, together with one cornea sample, were also analyzed using FAT method. The cornea sample was too firm to process for RNA analysis and was only detected using FAT. The results from all samples are summarized in Table 1.

For anti-mortem diagnosis, saliva samples gave the highest positive rate (32%), followed by CSF samples (around 21%). All 14 urine samples tested negative. Most notably, 9/85 (10%) serum (or blood) samples tested positive for viral RNA. Our results thus provide a new supplementary method to the recommended sampling procedure for suspected cases of rabies.

In the post-mortem diagnoses, the brain samples from all 23 cases tested positive. Neck skin tissues were available for two of the cases and these also tested positive. Only one cornea sample was available and this tested negative, despite a positive result from the brain sample.

The chi-square test showed that there was a significant different between positive test rates for different types of anti-mortem specimen (*P* < 0.001). Pairwise comparisons also showed a significant different between saliva and urine tests (*P* = 0.01) and between saliva and serum tests (*P* < 0.001).

### Laboratory-confirmed cases

A total of 65 of the 164 clinically suspected cases had a laboratory-confirmed diagnosis. Only one specimen was available for 114 suspected cases and two or more specimens were available for 50 suspected cases. Of the confirmed cases, 41 had only one available specimen and 24 had two or more available specimens. Of the 40 cases that had a positive anti-mortem diagnosis, 25 were confirmed by post-mortem diagnosis.

### Effect of sampling time

Data for 18 cases (with 40 samples) confirmed by anti-mortem diagnosis, with complete or partial clinical information, are listed in Table 2. It’s clear that saliva samples were tested earlier and more easily than CSF samples. Saliva samples from Case 1 and 7, collected on day 2 (post-onset of symptoms), tested positive. Only two CSF samples (Case 3 and Case 7) tested positive and, in each case, the saliva sample also tested positive. Case 4 and 10 showed the typical characteristics of intermittent shedding in saliva samples.

**Table 2:**
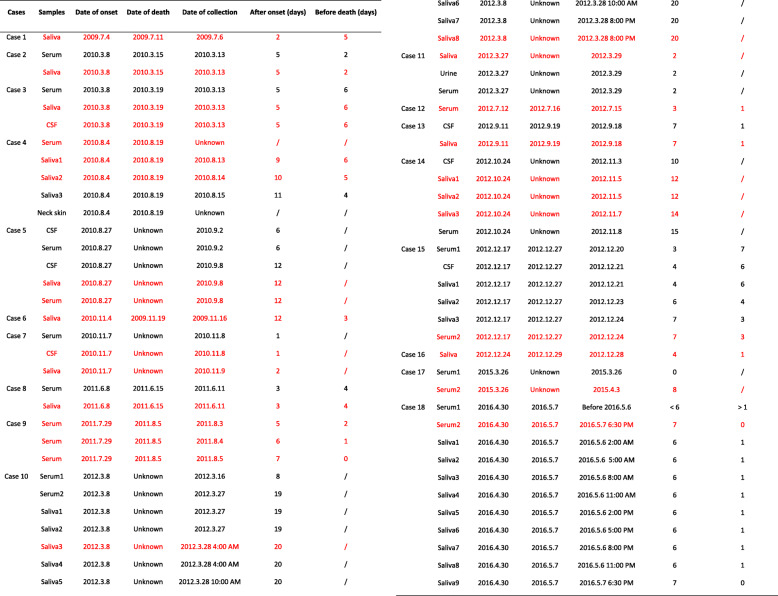
The detailed sampling data of the 18 confirmed cases by anti-mortem diagnosis (Red samples mean the positive results)

/ means not applicable

### Positive serum samples for viral RNA detection

Although the WHO only recommends collection of serum for viral antibody tests, we found that 10.6% (9/85) serum samples tested positive using RT-PCR (Tables 1 and 2). Seven cases were confirmed using only serum samples, another two cases were confirmed using both serum and saliva samples (Case 4 and Case 5). In the final days of life, serum samples were very sensitive and always tested positive during 0–3 days before death (Case 10, 12, 15 and 18). In Case 9, each serum sample collected in the last 3 days before death tested positive for viral RNA. Serum samples from the late stage of disease in four cases (Case 5, 15, 17 and 18) were positive, whereas serum samples taken earlier in the disease course tested negative.

## Discussion

Although viral detection is critical for disease surveillance and control, more clinically suspected cases of rabies are reported than laboratory-confirmed cases. In the absence of a history of exposure or typical symptoms, however, a diagnosis of rabies on clinical grounds alone may be difficult and often unreliable [8], and laboratory testing should be used to confirm the diagnosis [8].

The gold standard for confirmation of rabies is demonstration of viral antigen by FAT on brain tissue obtained post-mortem [8]. However, obtaining brain tissue after death remains a challenge and is rarely performed in China or many other countries [11, 16]. Despite significant developments in laboratory techniques, ante-mortem diagnosis of human rabies is fraught with difficulties. Although a validated positive ante-mortem test result is indicative of rabies, a negative result does not necessarily rule out a diagnosis of rabies in all cases, which is a major limitation of ante-mortem testing [8]. A combination of several tests on multiple clinical samples, with serial sampling whenever feasible, is therefore recommended to increase the accuracy of ante-mortem diagnosis [11].

Nucleic acid amplification techniques, such as RT-PCR, on saliva and CSF are increasingly being used for ante-mortem diagnosis of rabies. Our study shows that saliva RT-PCR provides the earliest and most sensitive positive diagnostic test (Tables 1 and 2). The typical characteristic of intermittent viral excretion in saliva samples is also evident in our study, which means that we strongly recommend collecting three or more serial daily saliva samples per patient.

The WHO and other experts recommend that serum samples should be used for anti-mortem diagnosis, but only used by measuring viral antibody [8, 16, 17]. Importantly, our study shows that serum or blood samples collected in the last 3 days of life can also be used to measure viral RNA. This means that serum samples, as well as other bodily fluids, such as saliva and CSF, can be used to detect viral RNA for anti-mortem diagnosis. We found that almost all the serum samples that tested positive were collected in the last 3 days of life (Table 2). Serum samples are easier to obtain than CSF samples, or even saliva samples in some situations at the end of life. Collection and analysis of serum in the late course of the disease will significantly increase the accuracy of anti-mortem diagnosis. Because of this, we have provided feasible sampling methods [18] and modified our “National Surveillance Project for Human Rabies”. The new guidance for front-line staff in local CDCs, which adds the collection and testing of serum from the end period of survival, will improve our surveillance and laboratory diagnosis of human rabies. We also hope that our research will provide another option for other countries facing similar rabies problems.

This study was limited to the samples of human cases. More than 2/3 cases only were collected one specimen or a kind of tissue, and multiple samples from one case with detailed clinical information will better support our conclusions.

## Conclusions

Serum or blood samples collected in the last 3 days of a patient’s life can be used to measure viral RNA. We recommend that serum samples should be collected and used for anti-mortem diagnosis of rabies by measuring viral RNA since this will improve the positive test rate in human cases.

## Data Availability

The data were already showed in this study.

## Abbreviations

CDCs: Centers for disease control and prevention
CSF: Cerebrospinal fluid
FAT: Florescent antibody Test
RT-PCR: Reverse transcription-polymerase chain reaction
WHO: World Health Organization
